# Explaining Siewierska’s generalization

**DOI:** 10.1007/s10828-021-09124-6

**Published:** 2021-04-29

**Authors:** Peter Hallman

**Affiliations:** Austrian Research Institute for Artificial Intelligence, Freyung 6/6, 1010 Vienna, Austria

**Keywords:** Double object alternation, Passive, Object symmetry, Inherent case, German

## Abstract

This article presents an explanation for a cross-linguistic gap observed by Anna Siewierska: morphologically unmarked indirect objects may alternate with prepositional marking in what is sometimes called a ‘dative’ or ‘prepositional-dative’ ditransitive frame, but never with actual dative case marking. ‘Dative’, to the extent it alternates with accusative, is always expressed as a preposition. I show firstly that German, which has a robust dative case paradigm, also displays a double object alternation in which the erstwhile dative DP occurs in a prepositional phrase, meaning both accusative (in English) and dative (in German) indirect objects alternate with prepositional encoding. I construct an analysis in which the the indirect object may be generated as either a DP (which receives dative in German and accusative in English) or a PP in the same theta position. This characterization of the double object alternation does not admit an alternation between dative and accusative case on the indirect object, capturing Siewierska’s generalization. The analysis also extends to ‘symmetric’ passive languages, in which either object in the double object construction can be raised to subject in the passive. Some current perspectives on this phenomenon make such languages exceptions to Siewierska’s generalization, but not the analysis proposed here.

## Introduction

This article seeks to explain a typological pattern observed by Siewierska (1998) to the effect that although accusative encoding of an indirect object often alternates productively with prepositional encoding, as in the English double object alternation, it never alternates with dative case encoding. I add here based on an examination of German double object constructions that although dative does not alternate with accusative, it does alternate with prepositional encoding, like accusative does in English. I present a syntactic analysis of this cross-linguistic gap that also addresses a potential challenge to Siewierska’s generalization presented by object-symmetric languages, that is, languages in which either object may be raised to subject in passive constructions. The resulting analysis accommodates both symmetric languages as well as asymmetric languages of both the German type (with a dative indirect object) and the English type (with an accusative indirect object), and identifies the parameters that distinguish these types.

## Setting the stage

I use the phrase ‘double object frame’ to refer to a verb complement frame consisting of two direct DP (‘Deteminer Phrase’) arguments, as in (1). ‘Direct’ here means not introduced by any adpositional material such as a preposition. Both objects in the English double object frame are morphologically unmarked, except as pronouns, where they are morphologically distinguished from subjects in the paradigm referred to as ‘accusative’. Following Harley (1995, 1997, 2002, 2012), Harley and Jung (2015), Beck and Johnson (2004), Beavers (2011) and others, I assume such constructions are causative alternants of an underlying possessive predicate and refer to *Mary* as the agent, *the collector* as the recipient, and *the pictures* as the theme. 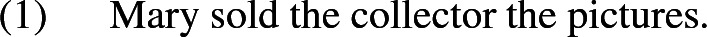


In this construction, the first nominal constituent following the verb is promoted to subject in the passive. 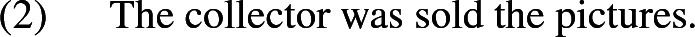


I use the term ‘periphrastic’ to refer to the alternant of the double object frame in which the recipient occurs in a prepositional phrase headed by *to*, as shown in (3a), and the theme promotes to subject in the passive, as shown in (3b). This frame is periphrastic in the sense that the recipient is encoded as such by an adposition. 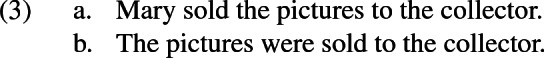


Many languages display a double object frame similar in form to English except that the two objects are differentially case marked. In German, as a case in point, the recipient argument receives dative case while the theme argument receives accusative case, as shown in (4). The subject receives nominative case. In German, the case of a DP is primarily reflected in the morphological form of the determiner in D. I follow the convention in German linguistics of citing examples in the form of a subordinate clause, to prevent alternations in grammatical function from being confounded with topicalization, which is largely limited to root clauses. 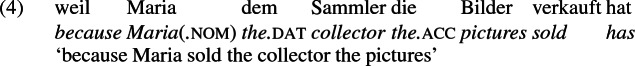


Passivization does not affect dative case, nor does it affect the canonical word order, as (5) shows. The recipient still canonically precedes the theme in the passive, but the theme functions as the subject in that it receives nominative case and controls agreement on the finite verb, visible in (5) as the plural inflection *-en* on the auxiliary *werden* ‘become’, a dedicated auxiliary for verbal passives in German (den Besten 1985, 1989; see also Thráinsson 1979; Andrews 1982, 1990; Zaenen et al. 1985; Schütze 1997 on similar configurations in Icelandic). 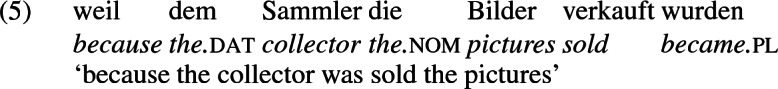


The recipient argument surfaces with dative case in German but accusative case in English. Across languages, therefore, dative is found in some languages in contexts where accusative is found in others. Yet, Siewierska’s generalization, sketched briefly in Sect. 1, dictates that dative never alternates with accusative within a language. Why should this be so? What parameter is at work and what syntactic mechanisms enforce it? I begin addressing this issue by describing Siewierska’s generalization in more detail.

## Siewierska’s generalization

In a sample of 270 languages, Siewierska (1998) identifies 38 that exhibit a clearly identifiable alternation between a double object frame and what she calls an ‘oblique’ frame, as illustrated by the English pair in (1) and (3a). But among the 270, she remarks, “no language which has dative marking of recipients, i.e. marking which does not double up as either allative or some type of locative marking, exhibits alternative patient-like encoding of recipients in ditransitive clauses” (p. 180). This means that in every language in which a recipient participates in an alternation between patient encoding and oblique encoding, the oblique encoding is an allative (i.e., ‘to’) or locative (i.e., ‘at’) preposition, never a dative case paradigm distinct from the morphemes that encode allative or locative meaning in the language in question.

This means that there is no language in which a dat-acc case frame like German alternates with an acc-acc frame like English, as illustrated by the paradigm in (6). In (6a), repeated from (4), dative marks the recipient argument, and this argument cannot be encoded accusative (6b), nor raised to subject in the passive (6c) (compare with English (1) and (2) respectively). According to Siewierska, neither (6b) nor (6c) are grammatical in any language in which (6a) is grammatical (where dative is characterized by being differentially marked from accusative and unaffected by passivization, as (5) shows).
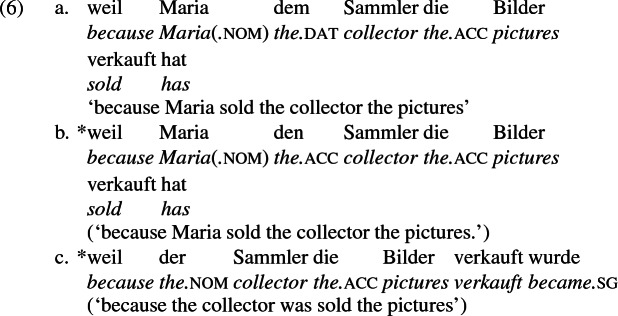


‘Patient-like’ encoding refers to the syntactic and morphological behaviour typical of patients (themes in my terminology), including morphological accusative case (typically unmarked in the languages Siewierska surveys) and the potential to raise to subject in the passive. Siewierska’s generalization says that dative does not alternate with accusative. But accusative alternates with what I call ‘periphrastic’ (i.e., prepositional, allative or locative) encoding. Siewierska concludes that periphrastic encoding in languages like English, i.e. *to*-phrases, which alternate with accusative, does not correspond to dative in languages like German, which does not alternate with accusative. Siewierska continues her remarks cited above with the conclusion: “Thus it appears that the term dative-shift is truly a misnomer” (p. 180). ‘Dative shift’ is a common term for a transformation that relates the periphrastic frame in (3a) to the double object frame in (1). On the basis of her typological generalization, Siewierska rejects the idea that allative *to*-phrases in English are on some level on par with dative case, encoding dative in a language without a dative inflectional paradigm. Rather, the English ‘double object alternation’ (the alternation between (1) and (3a)) does not involve dative case in either frame. Siewierska does not reject the notion that (1) is derived from (3a), she just emphasizes that it is wrong to refer to it as ‘dative shift’, since the *to*-phrase in (3a) is not dative. By the same token, it is incorrect to refer to the periphrastic frame in (3a) as the ‘dative’, ‘prepositional-dative’ or ‘*to*-dative’ frame.

I have more to say about the derivational relatedness of the periphrastic and double object frames in Sect. 4. The following section discusses the double object frame in German in more detail and seeks to demonstrate that in German, the dat-acc double object frame also alternates with a periphrastic frame, like in English. This observation reinforces Siewierska’s point that English *to*-phrases are not on par with dative DPs in German. Rather, they are on par with PPs in the German periphrastic frame. Section 5 then presents a syntactic analysis of the relation between the two ditransitive frames in German and English that has Siewierska’s generalization as a consequence. Section 6 looks at ‘symmetrical’ double object languages, in which either object may raise to subject in the passive. Some analyses of these languages make them exceptions to Siewierska’s generalization, but I show that on the analysis developed in Sect. 5 they are not. Finally, Sect. 7 considers the consequences of the analysis proposed here for a broader set of data in German and beyond.

## The double object alternation in German

As mentioned previously, double object constructions in German exhibit a similar canonical word order to English but mark the recipient argument in the dative case, and this case appears to be syntactically inert, in the sense that it cannot be shed in the course of passivization. On the other hand, many double object verbs in German exhibit an alternation with a periphrastic frame headed by the preposition *an* ‘at’, which assigns accusative case to its object. I present a number of examples below to impress upon the reader that the alternation is reasonably productive, though it has not been described as a double object alternation in German to my knowledge. These examples are constructed for the purpose of illustrating the phenomenon (a few based on attested examples but modified for parallelism). All have been confirmed by native German speakers, who agree that the sentences below are all grammatical, though the two frames may prefer different information structural contexts (see Bresnan et al. 2007; Rappaport Hovav and Levin 2008; Bresnan and Nikitina 2010 on this matter in English). Some of the examples contain hyphenation that does not reflect standard orthography, but is added to facilitate glossing. 
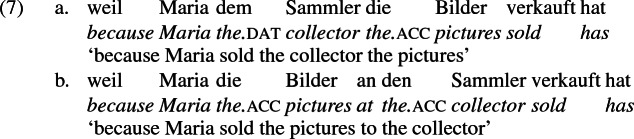

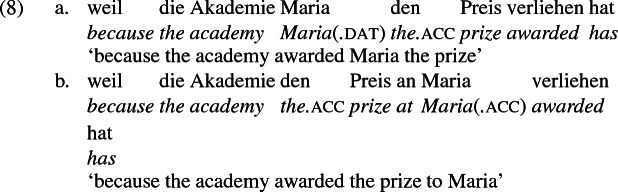

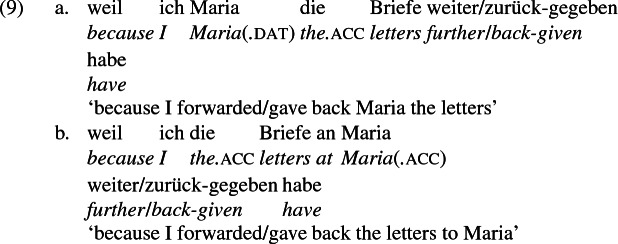

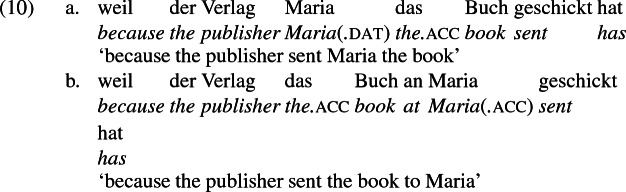

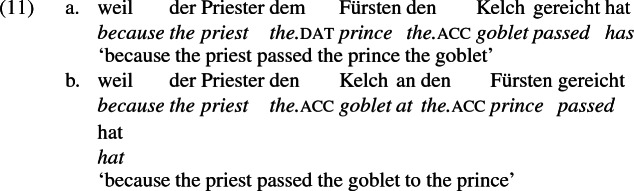

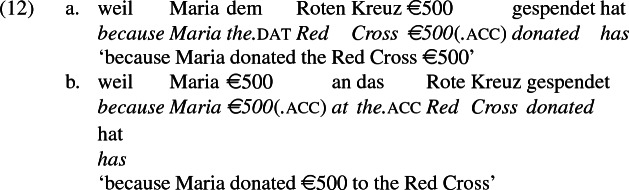

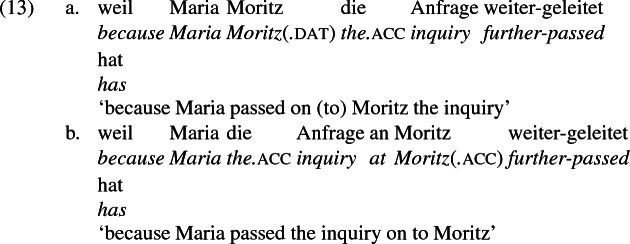

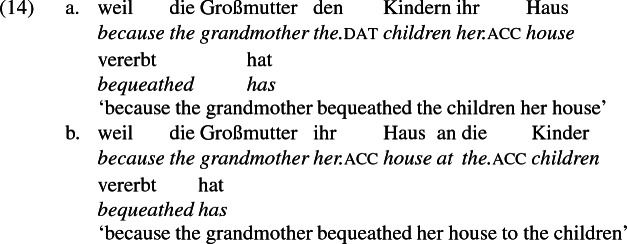

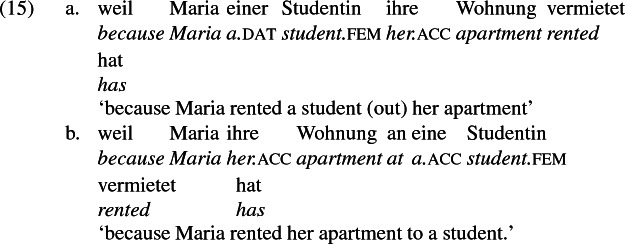

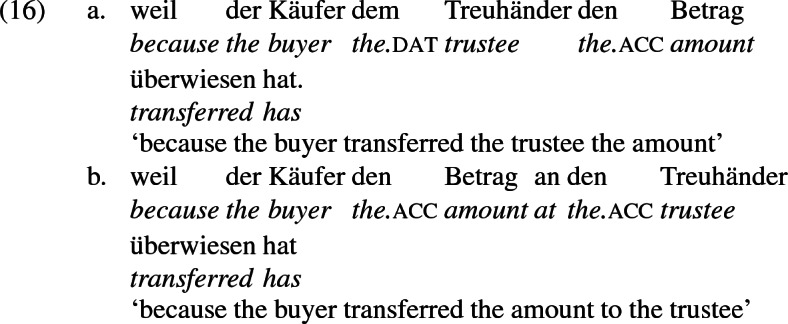

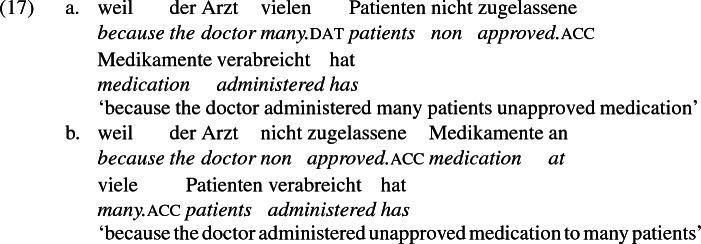

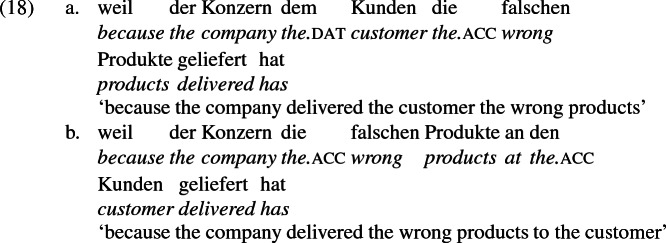

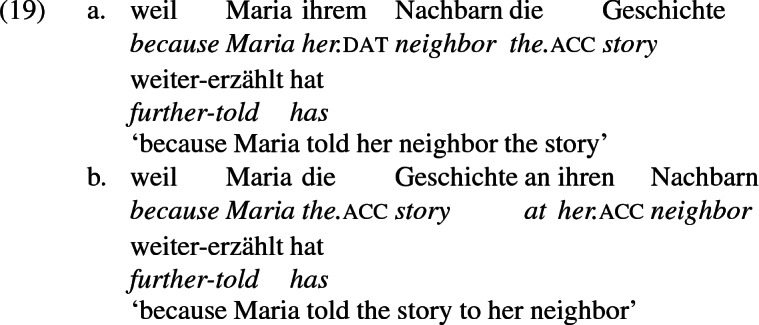

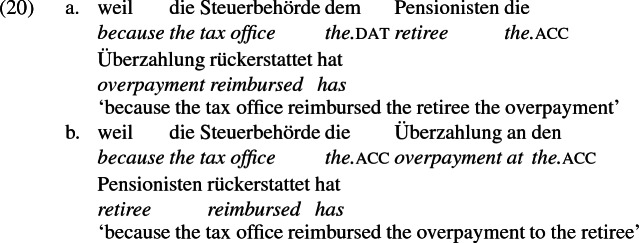

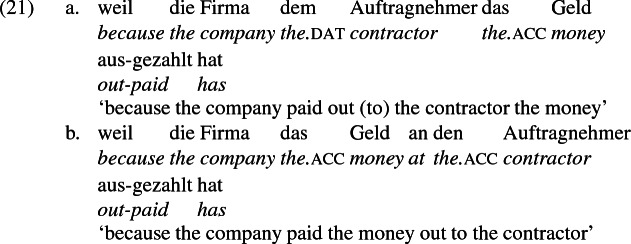


Not all verbs with a recipient argument admit both frames. Some, such as *geben* ‘give’ and *schenken* ‘gift’, i.e., ‘to give as a gift’ exclude the periphrastic frame, as (22) illustrates. The fact that *geben* excludes the periphrastic frame has probably obfuscated the productivity of the alternation in German.^1^
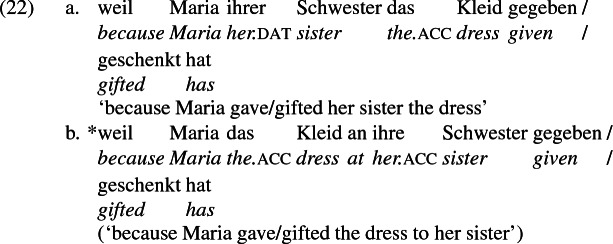


Crucially for the present purposes, when both complement frames are available to a given verb, they show two salient characteristics of constructional relatedness that indicate that they are distinct surface representations of the same underlying argument structure. One such characteristic is that the dative DPs in the a-examples in (7)–(21) are in complementary distribution with the PP counterparts in the b-examples, as (23) illustrates. This suggests that the dative DP and the PP counterpart are on some level the same argument, which therefore cannot be expressed twice in the same context. 
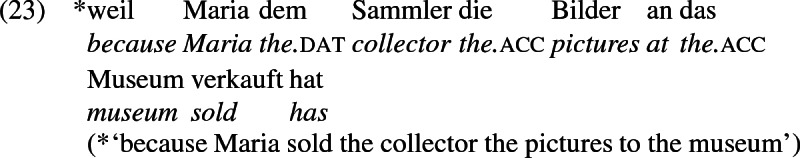


The other characteristic of constructional relatedness is that the dative arguments in the a-examples above are subject to the same selectional restrictions as their PP counterparts in the b-examples. Whenever both frames are available, the restrictions on one are found in the other. Specifically, the recipient argument must be a potential possessor. It need not necessarily be animate, but if inanimate, it must be capable of possessing the theme. Because a museum can possess pictures, *Museum* can occur both as a dative and periphrastic recipient of *verkaufen* ‘sell’, as (24) shows. But since the North Sea cannot be understood as a possessor of pictures, it cannot occur as a dative object in (25a), nor as a periphrastic object in (25b). 
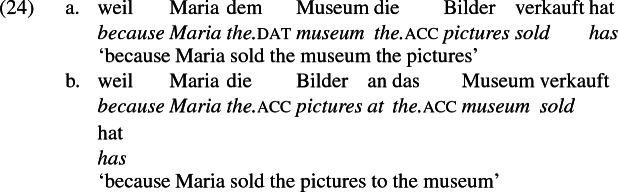

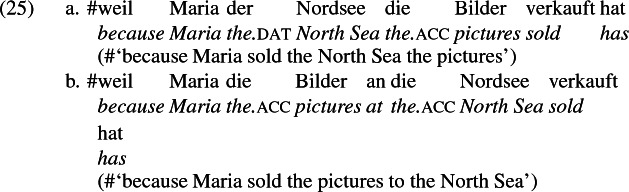


In principle, the phrases *das Museum* ‘the museum’ and *die Nordsee* ‘the North Sea’ can name locations, as in the English *I’ll see you at the museum* or *We spent the summer at the North Sea* and their German counterparts. But the fact that the former but not the latter is a possible indirect object of *sell* indicates that these phrases do not function as location arguments in (24) and (25), but rather as recipient arguments, for which *das Museum* ‘the museum’ is qualified but not *die Nordsee* ‘the North Sea’. That is, the dative argument of *verkaufen* ‘sell’ is a recipient (a kind of possessor). The contrast between (24) and (25) shows that the periphrastic argument is a recipient as well. The dative and periphrastic arguments of *verkaufen* bear the same theta role.

A closer look at the preposition *an* ‘at’ reinforces the claim that in the b-examples above, *an* marks the same theta role as dative marks in the a-examples, meaning the pairs are alternative encodings of the same underlying argument structure. In certain periphrastic constructions, *an* indeed seems to have a locative use. But in this use, it does not alternate with dative in the double object frame, reinforcing the point that alternating *an* marks a recipient and is constructionally related to dative.

Rappaport Hovav and Levin (2008) argue that certain *to*-phrases in English are ambiguous between a recipient role and a location role, but that this ambiguity is a lexical idiosyncrasy of certain verbs and not the basis of the alternation between the double object frame and the periphrastic frame. The paradigm case of such a verb is *send*. The *to*-phrase associated with *send* can contain either a human (26a) or non-human (26b) DP, but, as Green (1974), Oehrle (1976) and others point out, only the human *to*-phrase alternates with a double object frame, as (27) demonstrates. (27b) is only acceptable if *London* is construed as a personification implying a human referent, not the location so named. (26b) is not subject to this restriction. 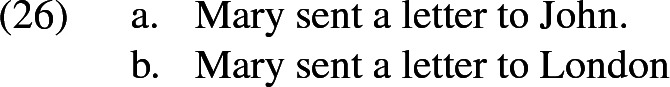

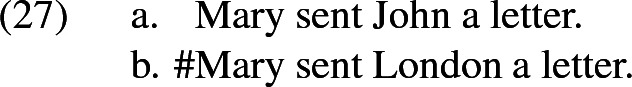


On the basis of this and other observations, Bowers (1981) and Hallman (2015) analyze (26a) as derivative of (27a). Example (26b), however, is a basic locative construction describing change of location, rather than change of possession. The expression *send X to Y* is therefore structurally ambiguous. It may be parsed as a locative construction in which *X* is the theme and *Y* a location, or as a change-of-possession construction syntactically related to *Send Y X*, where *Y* is the recipient and *X* the theme.

Data from German support these conclusions and reinforce the claim that the *an*-phrase in the examples above is not a locative phrase but a recipient phrase syntactically related to the corresponding dative DP. The German counterpart to (27b), shown in (28a) is as infelicitous as its English counterpart; it is only sensible if *London* is construed as a personification implying a human referent (i.e., the personnel in London). Crucially, the periphrastic counterpart with *an* is infelicitous in exactly the same way, supporting the view that the dative phrase in (28a) and the periphrastic phrase in (28b) bear the same theta role. Since the recipient theta role is incompatible with a city name in the dative, it is incompatible with it in the *an*-phrase as well. 
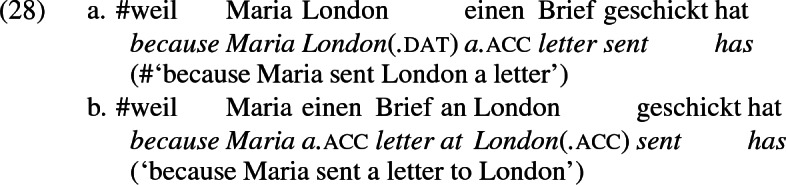


In German, *schicken* ‘send’ may also be construed locatively with a place-name argument, but then the place name occurs in a prepositional phrase headed by *nach*—allative ‘to’ in German, as (29a) illustrates. The preposition *nach* is not compatible with a human object, as (29b) shows. See Levin (2008) for similar remarks on Russian and Hebrew, and Citko (2011) on Polish. 
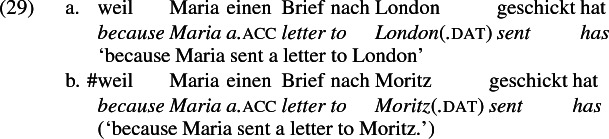


A comparison of German and English supports the idea that the English phrases *send to Moritz* and *send to London* exemplify different constructions, one a change-of-possession construction and the other a change-of-location construction. If the double object frame encodes change-of-possession, then (28a) is ruled out because London cannot function as a recipient. If (28b) is ruled out because the *an*-phrase encodes the same theta role as dative in the double object frame, then an argument that is acceptable as a dative recipient should be acceptable in place of *London* in (28b). The pair in (10) shows that this is so, reformulated in (30) parallel to (28), 
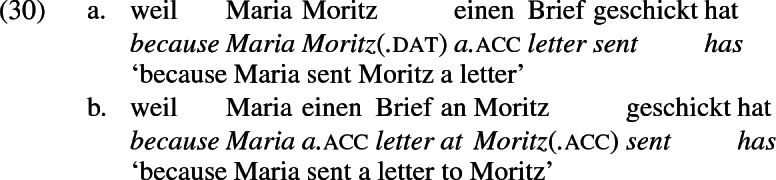


Since *schicken* ‘send’ may be construed as a locative structure, location-denoting expressions built with the preposition *an* ‘at’ may occur with the locative construal of *schicken* ‘send’, leading in a limited range of contexts to a situation similar to the ambiguity in English in the interpretation of *to* as recipient encoding or location encoding. For example, the phrases *at the beach* and *at the front* are constructed in German with *an*, as the a-examples below show. And *an* shows up in constructions describing movement to the location so named, as the b-examples show. 
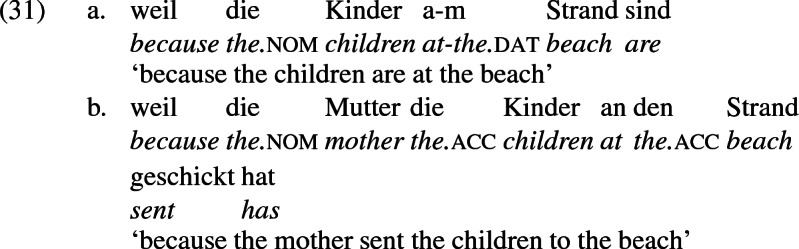

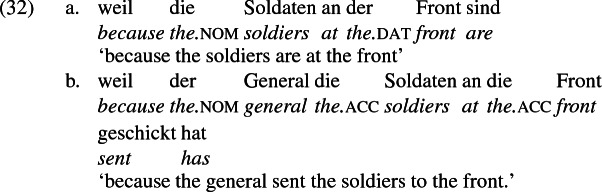


But location-denoting phrases that are not built with *an* ‘at’ do not use *an* in change-of-location constructions with *schicken* ‘send’ either. 
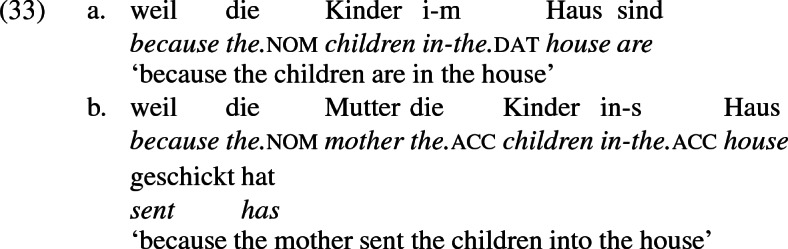

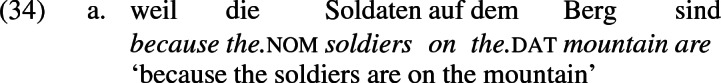


As a general rule, then, a locative preposition can be used in a change-of-location construction describing movement to that location. Occasionally, this preposition is *an*, as in (31) and (32) , but not generally, as  (33) and (34) show. On the other hand, *an* is used to mark recipients generally, as in (35a), independently of whether a locative construction can be constructed in the same terms (35b). 
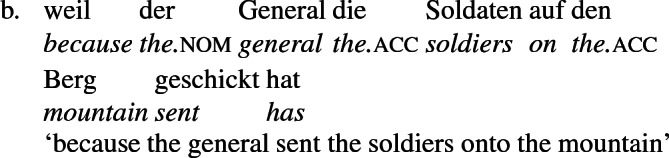


These facts suggest that recipient *an*-phrases are syntactically distinct from locative *an*-phrases, and only the former are constructionally related to dative recipients. This conclusion of course raises the question of what the relation is. In the following section, I present an analysis of the double object alternation in German and English that addresses this issue and captures Siewierska’s generalization.

## Analysis: external and internal voice alternations

I assume that transitive verbs are syntactically complex along the lines postulated by Chomsky (1995), Harley (1995), Kratzer (1996) and others. The agent is base generated in the specifier position of a light verbal head ‘little-v’, whose complement is the projection of a verbal root whose specifier hosts the theme, as illustrated in (36b) for the transitive sentence in (36b). Following Chomsky (2000, 2001, 2004) and others, I assume the Agree relation extends from a probe to the closest potential goal in the probe’s c-command domain and that nominative case is the morphological reflex of the Agree relation between T[ense] and its goal, as signified by the arrow in (36b). 
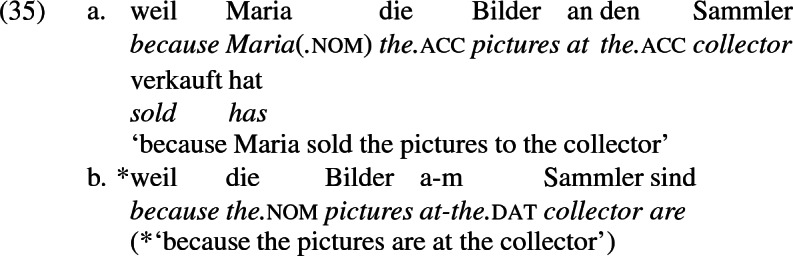


In English, Agree typically goes hand in hand with raising of the goal to the probe, putting the subject in [spec,TP], unless a placeholder subject fills the subject position in its stead as in existential-*there* constructions. In German, the nominative-marked subject may remain in situ (den Besten 1985; Haider 1993, 2006; Wurmbrand 2006). The arrow in the tree above represents the Agree relation licensing nominative on the subject; the feature [nom] under T represents the nominative licensing potential of T. Movement (obligatory in English, optional in German) is not shown. Also, I place the verbal root in V, though it might be associated post-syntactically with the v-V complex formed by head movement at a later stage in the derivation, along the lines of the Distributed Morphology theory (Halle and Marantz 1993; Harley 2007, 2012). The tree above depicts the base structure for the German sentence in (36b), but it is intended to capture the base structure for its English translation there, too, modulo superficial differences in the directionality of headedness, DP raising, verb movement and the expression of tense (perfect in German vs. preterit in English). The CP layer containing *weil* ‘because’ is not shown, since it is not involved in case- or theta-assignment.

Little-v is typically analyzed as a probe licensing accusative case on an object (Chomsky 2000 and elsewhere; see Ura 1996; Collins 1997; McGinnis 1998 on double object constructions in particular). Nothing I say about German or English in this section conflicts with this view. However, the facts about symmetric object languages that I discuss in Sect. 6 point to the necessity for a theory of accusative case which divorces it from any specific syntactic locus. I anticipate the conclusions of that discussion by positing a default accusative-assigning mechanism: if a predicate-internal DP reaches PF (the surface level of representation ‘phonological form’) with an unvalued case feature, that feature is valued to [acc]. Unlike accusative, nominative case is licensed by a specific probe, T, under Agree (as is dative, as I describe below).

Roberts (1987), Bruening (2013) and others argue in detail that English *by*-phrases in the passive encode the external argument of the corresponding active, based on selectional regularities similar to what I discussed above for dative DPs and *an*-phrases in German ditransitive constructions. They conclude that *by*-phrases adjoin to vP and that *by* is semantically transparent—it merely passes the external theta role to its DP complement. Hasegawa (1988), Goodall (1997), Mahajan (1994), Collins (2018) and Angelopoulos et al. (2020) analyze these selectional regularities as the result of a shared base position: the *by*-phrase is base generated in [spec,vP] in passives, just where the external argument DP is generated in actives. I show below that the latter view offers some mileage in pinning down the source of Siewierska’s generalization, once we view the double object alternation as an ‘internal’ passive construction, as do Perlmutter and Postal (1984) and Larson (1988), though my analysis differs from theirs in important details.

I propose therefore that the base structure for both the German passive sentence in (37a) and its English translation there (again modulo certain systematic differences) is that diagrammed in (37b), where, since the external argument receives case internal to the *von/by* phrase, it is not visible to the Agree relation extending from T, which finds and assigns nominative case to the theme instead. This nominative DP raises to [spec,TP] optionally in German and obligatorily in English. 
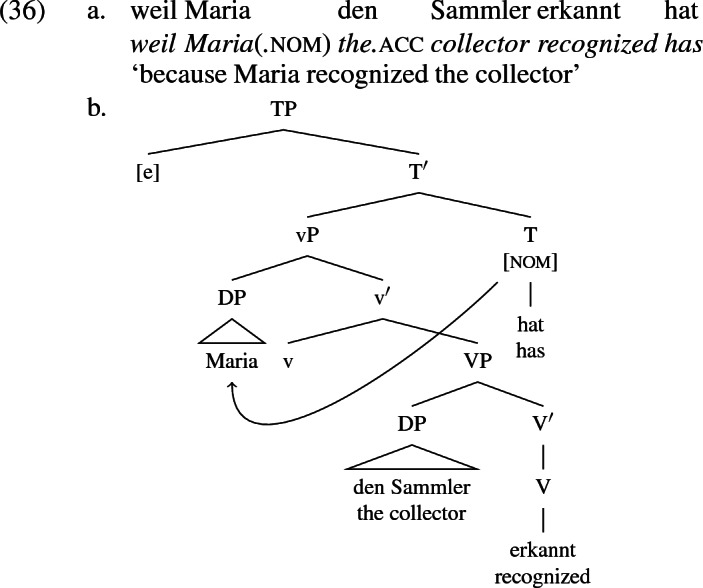


According to this analysis, the main difference between active and passive structures is that the agent is generated in a PP in the latter. The transference of nominative case to the next highest DP is a consequence: because the agent is case-valued internal to the PP, it is not a potential goal for the nominative probe, which therefore probes past it and finds the theme. This PP can be elided, generating the ‘short passive’ (the passive without the *by*-phrase). On this analysis, passivization correlates not with withdrawal of objective Case but with demotion of the agent into a PP. As a consequence of demotion of the agent, nominative moves down to the next highest DP. This DP does not receive accusative Case, not because its accusative has been withdrawn, but because it has received nominative from T before the pre-spell-out point at which accusative is assigned by default. It is crucial to this analysis that T *must* agree with the highest DP in its domain if there is one,^2^ assigning nominative case to that DP. If Agree were optional, that DP could receive default accusative instead, being predicate internal, contrary to fact. In English, the nominative-licensed theme raises to the subject position [spec,TP], where it precedes the *by*-phrase. This word order is available in German as well, as is the word order generated in (37b). I turn to the double object construction in English and German with these tools in hand.

Following Bowers (1993), Marantz (1993), Collins and Thráinsson (1996), Bruening (2001), Harley (2002, 2012), Harley and Jung (2015), and others, I attribute the the basic predicate structure in (38b) to the double object frame shown in English (38a). This tree is like the monotransitive construction in (36b) except that a recipient argument is introduced in the specifier of ApplP (‘Applicative Phrase’), which occurs between vP and VP. In the active, the agent receives nominative from T as before. Both objects receive accusative case by the default rule discussed above. 
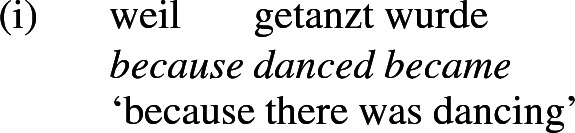


McGinnis (1998), Cuervo (2003b), Anagnostopoulou (2003), Woolford (2006), McFadden (2006) and McIntyre (2006) claim for a variety of languages that dative case is assigned to the recipient argument in its base position within ApplP. The notion that dative is assigned configurationally in change-of-possession constructions has a precedent predating the split-VP hypothesis in works by Fanselow (1987), Czepluch (1988), Wegener (1991) and others. Following this lead, I propose that German is fundamentally the same as English, except that the recipient in [spec,ApplP] receives dative case directly from Appl in the spec-head relation. That is, Appl assigns case to its own semantic dependent—the recipient. The agent receives nominative from T and the theme receives accusative again by default. 
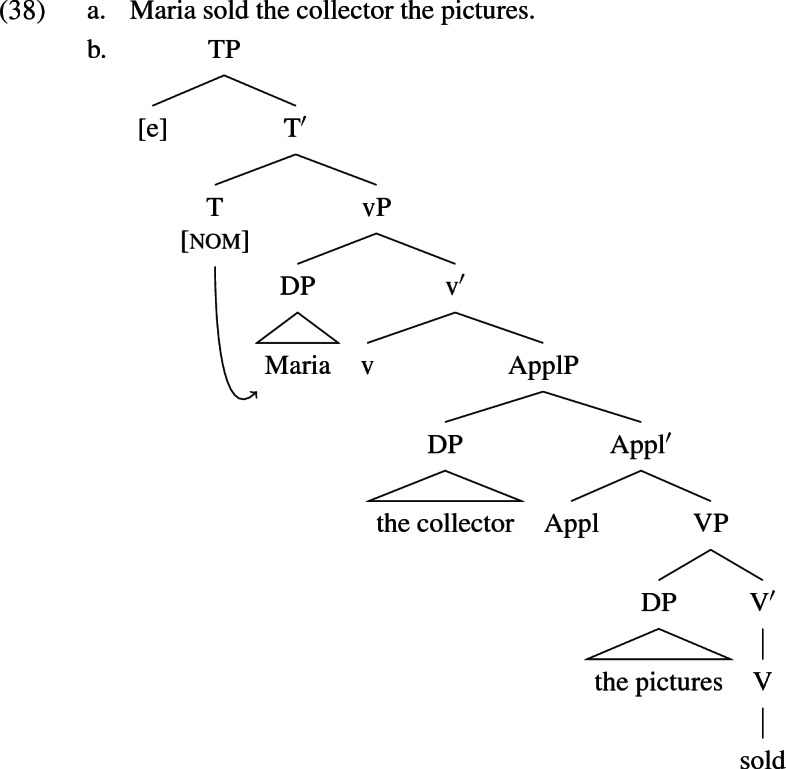


In the passive of (39a), mentioned in (5) and repeated in (40) below, the theme *die Bilder* ‘the pictures’ receives nominative case and controls agreement on the finite auxiliary *wurden* ‘became’. This means that the dative recipient *dem Sammler* ‘the collector’ does not function as an intervenor for the Agree relation between T and the theme that transmits nominative case. 
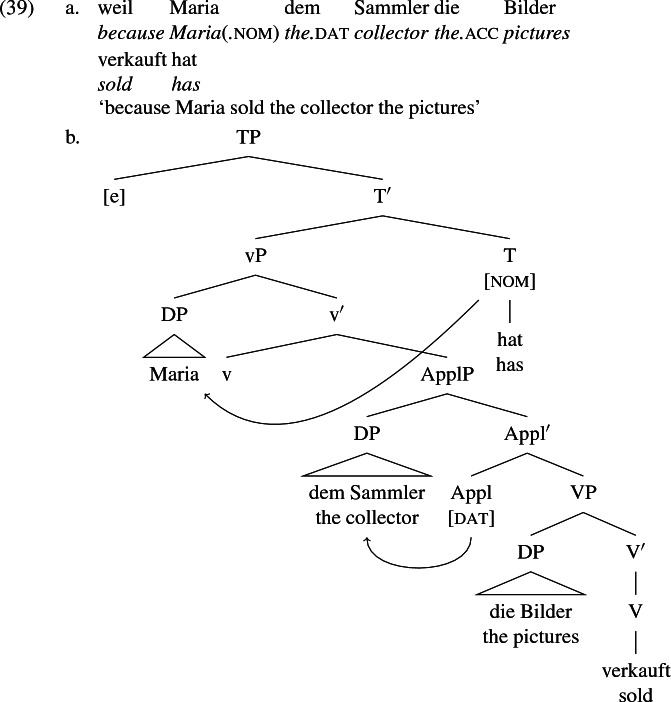


That the dative DP is invisible to the probe T is expected, since the dative DP is already case-valued by the time T is merged, much like the agent in the *von*-phrase in the passive, which receives case from *von* ‘by’. Dative DPs have been observed to induce ‘defective intervention’ effects in some languages, such as Icelandic (Chomsky 2000; Holmberg and Hróarsdóttir 2004). There, a dative DP does not disrupt case assignment to a lower DP but may disrupt transfer of agreement features from the lower DP to the case assigning probe. In German, however, dative DPs disrupt neither case assignment nor feature transfer and so are not intervenors for $\phi$-agreement chains in any sense (Broekhuis 2007). On the assumption that case-saturated DPs are not visible to $\phi$-Agree relations that emerge later in the derivation, neither the dative recipient nor the periphrastically encoded agent are eligible for case, which makes them transparent to the Agree relation between T and lower unsaturated DPs.

As described in detail in Sect. 4, the recipient argument in German can alternatively appear in a prepositional phrase headed by *an* ‘at’, in which case the recipient DP receives accusative case from *an*. The selectional regularities described there indicate that dative and periphrastic recipients bear the same theta role. Recall, too, that when we control for the ambiguity of the English periphrastic marker *to* with the homophonous allative preposition, the selectional regularities observed in German apply to English as well. That is, the grammaticality of *I sent the letter to London* does not militate against a uniform base structure for the double object and periphrastic frames (in spite of *#I sent London the letter*) if we allow for the possiblity that the DP-PP frame of some verbs (including *send*) is structurally ambiguous between a change-of-possession construction syntactically related to the double object frame and a locative construction unrelated to the double object frame. These structures are differentiated by the choice of preposition in German (*an* vs. *nach* or other allative preposition) but morphologically neutralized in English.

Drawing on the intuition expressed by Perlmutter and Postal (1984) and Larson (1988) that the double object alternation is a kind of ‘internal passive’, I model the double object alternation after the analysis of passive (or what one might call ‘external passive’) shown above in (37b). The recipient may be base generated in [spec,ApplP] either in the form of a bare DP, as shown in (39b) (dative in German and accusative in English), or in the form of a prepositional phrase (headed by *an* ‘at’ in German and *to* in English), as diagrammed in (41b). I assume that *an* and *to* in this usage are semantically vacuous, like their counterparts *von* and *by* in the external passive. The agent receives case under Agree from T, the recipient receives case from the preposition, and the theme receives default accusative in both languages. Here again, (41b) is intended to represent the base structure of both the German sentence in (41a) and its English translation there, and abstracts away from various surface differences between the two languages. 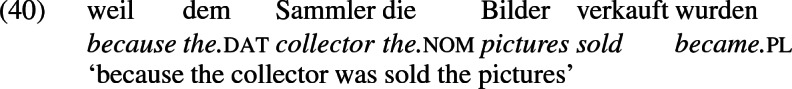


The word order generated in (41b) is a possible surface word order in German, as is the order in which the theme DP precedes the recipient PP. I assume the latter is derived by raising of DP above PP. Word order in German is subject to a variety of conditions including definiteness, animacy, quantifier scope and prosody (Diesing 1992; Büring 2001; Frey 2001; Heck 2001; Pafel 2005, among many others) which, according to the analysis in (41b), condition raising of DP above PP as well as other transformations. In English, the DP-PP order is obligatory. This suggests that what is optional in German is obligatory in English. I leave the examination of conditions on surface word order in German and their relation to the more restricted English pattern for another occasion.

The internal passive shown in (41b) may of course co-occur with the ‘external’ passive, so that both the agent and the recipient are expressed in PPs, as (42) illustrates. 
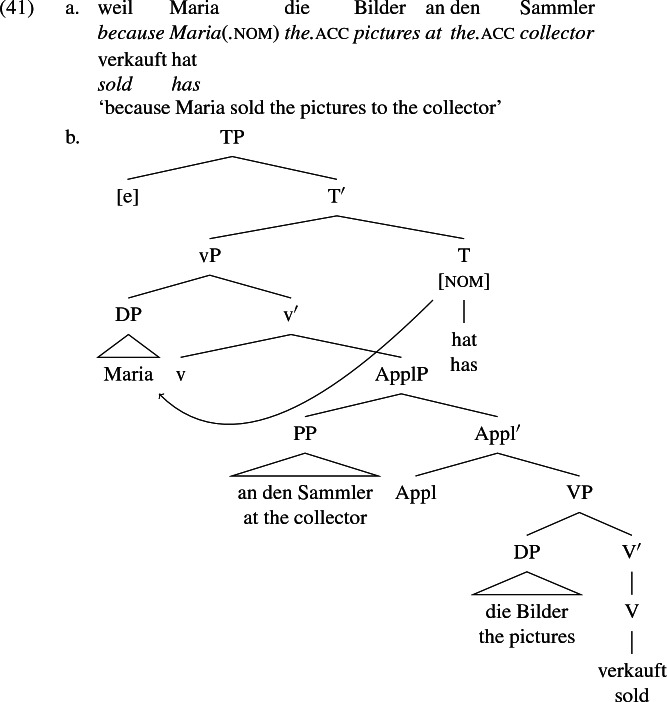


According to this analysis, German and English are uniform in terms of the structure underlying the double object frame and the periphrastic frame. These differ in whether the recipient is generated as a DP or a PP. This is just the distinction that differentiates the ‘external’ active and passive, in which the agent is base generated as a DP or a PP respectively. This common basis for change-of-possession constructions in the two languages is schematized in (43a), where the braces indicate that either DP or PP is generated in this position. Nothing I have said above militates against the conventional analysis of change-of-location constructions as structures in which a theme occurs in the specifier of VP whose complement is the PP designating the location, as schematized in (43b). The pairs in (7)–(21) display the structure in (43a) with the choice of DP and PP as specifier of ApplP in the a- and b-examples respectively. The change-of-location construction in (29a), on the other hand, displays the structure in (43b). In both structures, the ‘external’ passive arises by choosing PP in [spec,vP]. 
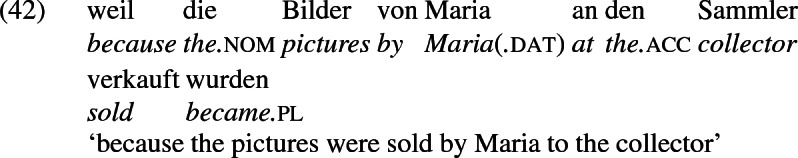


Another consideration that supports the distinction between (43a) and (43b) and the analysis of the b-examples in (7)–(21) (with *an*-phrases) as internal passives is that the *an*-phrases in these examples are systematically optional in German, just like *von* ‘by’-phrases in passives. As remarked above, a *von*-phrase hosting an agent may be dropped, to yield what is often called the ‘short’ passive illustrated in (44a). Recipient *an*-phrases share this property; ellipsis of an *an*-phrase represents a ‘short internal’ passive, illustrated in (44b). The possibility of dropping a PP is not, however, typical of locative PPs, as the unambiguously locative examples in (45) demonstrate. 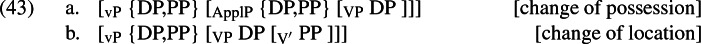

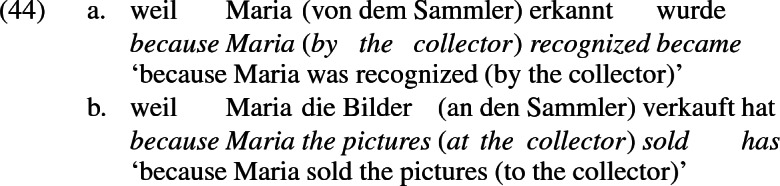


These observations reinforce the claim that recipient *an*-phrases are different from locative PPs and bear a resemblance to *von*-phrases hosting agents in the passive. This resemblance in turn reinforces the idea that the periphrastic change-of-possession frame is an internal passive, for which the double object frame is the corresponding internal active.

A reviewer of the present work points out that the *an*-phrases that I call recipients pattern with locative prepositional phrases in accepting directional modifiers like *direkt* ‘directly’. *Direkt* can modify a directional PP as in (46a) or an *an*-phrase naming a recipient as in (46b), modeled after (14b) above. 
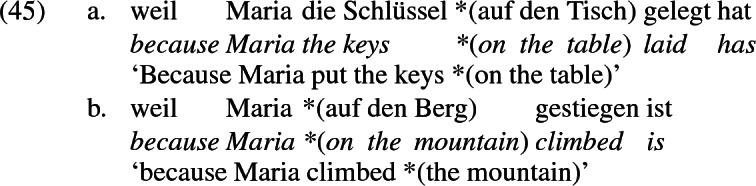


It is clear that *direkt* and similar words are PP modifiers; neither instance of *direkt* in (46) can be separated from the following PP preserving meaning. The literature on PP modifiers does not treat the meaning of modifiers of directional PPs in great detail. Zwarts (1997) claims that stative locative prepositional phrases denote sets of vectors—oriented paths extending from one point of reference to another—so that *above the door* denotes a set of vectors extending from the door upward. He claims that the modifier *directly* or *right* as in *directly/right above the door* restricts the set of vectors that *above the door* denotes to ones that are very short. But even the clearly locative example in (46a) does not seem to assert as part of its meaning that the path the groceries took to the refrigerator was short. Rather, it asserts that the groceries did not come to rest in any third location between their starting location (for example, in the grocery bags) and the refrigerator. This schema for the meaning of *direkt* in (46a) extends to (46b) with the modification that the latter describes change of possession rather than change of location. (46b) asserts intuitively that the house did not come into the possession of any third party between being possessed by the grandmother and by her grandchildren. This assertion is informative because one might have expected the house to come into the possession of the grandmother’s children first.

The remarks above point to the conclusion that *direkt* deals in alternatives, much like focus particles like *nur* ‘only’. *Nur* may also adjoin to a PP, as the examples in (47) show. 
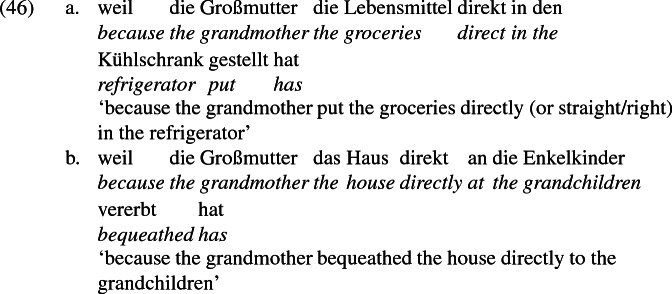


*Nur* does not have scope at the level of the PP, but rather, according to Rooth (1985, 1992, 1996) and others, at the level of the VP. There, it denotes a relation between the denotation of VP and a set *C* of alternatives to VP, asserting that if any member of *C* holds, it is the VP-denotation itself, i.e., no alternatives to VP are true. The PP-adjacent position of *nur* in (47) serves to focus-mark the DP in the PP (notated by the subscript *F*), which restricts the alternatives in *C* to those that differ from the VP denotation only in the value of the focus-marked position. Consequently, (47a) asserts that the grandmother put the groceries nowhere other than in the refrigerator, and (47b) asserts that the grandmother bequeathed the house to no one other than the grandchildren.

Below I sketch a parallel analysis of *direkt*, still assuming a causative semantics for change of location and change of possession, where little-v denotes the cause relation and the complement of little-v describes the caused state. A null be heads V in the state description [$_{\text {VP}}$ the groceries be [$_{\text {PP}}$ in the refrigerator]] and a null have heads the Appl+V complex in [$_{\text {ApplP}}$ [$_{\text {PP}}$ to the grandchildren] have [$_{\text {VP}}$ the house]] (recall that I claim that the preposition *to/an* is vacuous in change-of-possession constructions). In the surface structure, *directly* (=*direkt*) modifies a PP and focus marks its complement DP, as shown in (48). At LF, it modifies the result state description—VP in the locative construction, shown in (49a) and ApplP in the possessive construction, shown in (49b). 
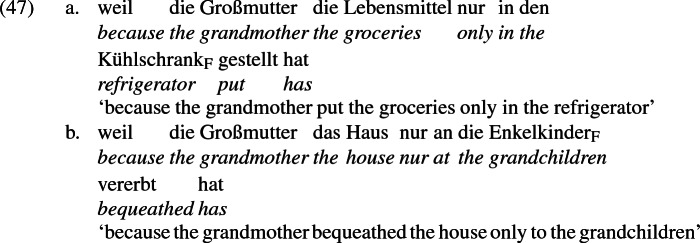

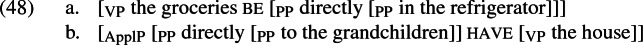


*Direkt* itself denotes a relation between a proposition $\phi$ (the denotation of VP/ApplP) and a set *C* of alternatives to $\phi$, and asserts that $\phi$ holds prior to any alternative in *C*, as defined in (50). As a result of focus marking, the alternatives for the locative construction in (46a) are ‘the groceries are in *x*’ for some place *x* and for the possessive construction ‘*x* has the house’ for some possessor *x*. The locative sentence in (46a) asserts, then, that the grandmother caused it to be the case that the groceries were in the refrigerator before being anywhere else, and the possessive construction in (46b) asserts that the grandmother caused it to be the case that her grandchildren had the house before anyone else had the house. The usual trajectory of inheritance dictates that the grandchildren would eventually come into possession of the house anyway, but we would have expected their parents to own it first. The use of *direkt* in (46b) denies this expectation. 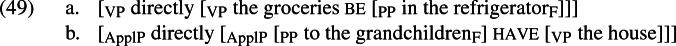


Again, the position of *direkt* adjacent to the PP in the surface structure only serves to mark the focused constituent, where the ‘gap’ occurs in the set of alternatives to the proposition argument of *direkt*. In all likelihood, this analysis of *direkt* requires some refinement. The aim of these remarks is to show that a purely vector-based analysis of *direkt* does not seem to be warranted even for the unambiguously locative example in (46a). Analyses of locative *direkt* (in phrases like *direkt über der Tür* ‘directly above the door’) where it restricts a set of vectors to short ones do not obviously carry over to (46a), where something more complex seems to be happening, specifically, something more akin to what is happening in focus particle constructions like (47a). An analysis of *direkt* that makes it a kind of focus particle extends readily to its use with recipient *an*-phrases in examples like (46b).

Returning now to Siewierska’s generalization, if a language were to display an alternation between an accusative and a dative recipient, it would mean that Appl assigns dative to its specifier optionally. In the absence of dative assignment, the specifier of ApplP would receive case from whatever mechanisms the language has at its disposal for the assignment of accusative. Siewierska observes that this does not happen; Appl cannot optionally withhold dative case to a DP specifier. Whether Appl assigns dative to a DP specifier or not is parametrically specified for the language. But this parameter is unrelated to the alternation between the double object frame and the periphrastic frame, which takes the form of an optionality in whether the recipient is base generated as a DP (with dative in German) or a PP. This alternation is an ApplP-internal instance of the active/passive alternation. The investigation of German above also reinforces Siewierska’s point that recipient *to*-phrases in English are not comparable to dative recipient DPs in German. Rather, they are comparable to *an*-phrase recipients in German. The alternation between the double object frame and the periphrastic frame is independent of the distribution of dative case. The grammatical properties in (51)–(52) summarize this analysis of German and English ditransitive constructions. Siewierska’s generalization results from the point in (52), that dative cannot be withheld. 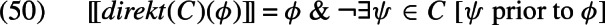

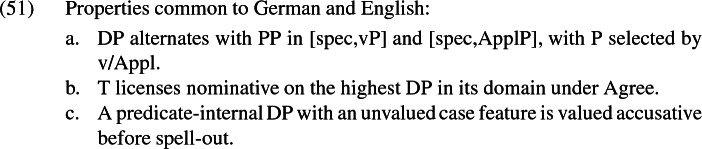


In the following section, I turn to ‘symmetric object’ languages. Case-based analyses of the phenomenon they represent make them exceptions to Siewierska’s generalization. I show that from the perspective of the analysis described above for English and German, this is not so.

## Symmetric double object languages

In some languages, when a double object construction is externally passivized (that is, the subject is demoted to PP and an object raises to subject in its place), either object may be raised to subject (Ura 1996; McGinnis 1998; Anagnostopoulou 2003; Bissell Doggett 2004; Haddican 2010; Haddican and Holmberg 2012, 2019; Holmberg et al. 2019). Norwegian and Swedish are a well-studied case in point, illustrated for Norwegian in (53) (Holmberg and Platzack’s 1995 example 7.69, p. 215). Some dialects of English behave in this way as well; see especially Haddican (2010). The phenomenon is often referred to as ‘symmetric’ passivization. 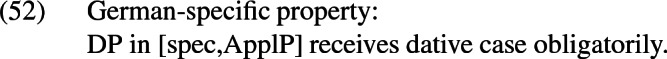


Haddican and Holmberg (2019) and Holmberg et al. (2019) claim that this pattern results from an optionality in the direction of case assignment by Appl. Appl may assign case upward to its specifier the recipient, represented by the solid arrow extending from Appl in (54), or downward to the theme in a lower position, represented by the dashed arrow extending from Appl in (54). In each case, little-v assigns accusative to the other argument; the solid arrow extending from v accompanies the other solid arrow, the dashed arrow the other dashed arrow. Case assignment is modeled as checking of an uninterpretable feature of the DP against an interpretable feature ‘[*i*Case]’ of the case assigning head. This tree amalgamates Holmberg et al.’s trees (28) and (29) (p. 689). 
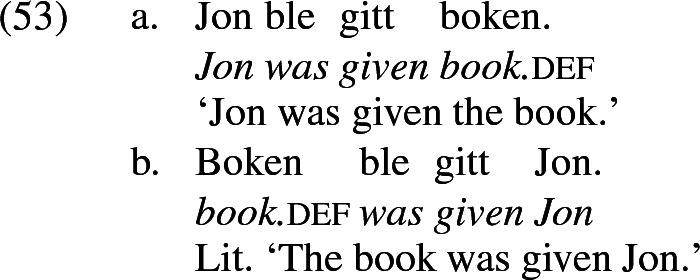


When Appl assigns case downward, it case-licenses the lower theme. Its own specifier, the recipient, receives case from little-v. Passivization involves withdrawal of the interpretable case feature from little-v, triggering promotion of the recipient to subject. This derives the pattern seen in (53a), also seen in standard English. In the other case, Appl assigns case upward to its own specifier, the recipient. The theme then receives case from little-v. Once again, withdrawal of little-v’s potential to assign accusative results in promotion of its erstwhile goal to subject, this time the theme, deriving (53b).

Somewhat similar analyses are proposed by Citko (2011) and Haddican and Holmberg (2014), which differ in that the case that Appl assigns upward in (54) is assigned downward by a higher head that occurs in between little-v and ApplP. On these accounts, too, the recipient’s case is able to be licensed locally by a head independent of little-v, so that little-v assigns case to the theme instead, paving the way for promotion of the theme to subject when little-v’s case assigning potential is withdrawn in the passive.

According to these analyses, the recipient’s case can be licensed at a derivational stage prior to merger of little-v, rendering the recipient inert as a potential goal for little-v. I refer to this situation as ‘local case assignment to the recipient’, meaning the recipient’s case assigner is more local to the recipient than little-v is. In the symmetrical languages, local assignment of case to the recipient is optional. When the recipient gets local case, little-v assigns case to the theme. When little-v’s case assigning potential is withdrawn in the passive, the theme raises to subject. When the recipient does not get local case, it gets case from little-v instead (however the theme gets case). Then, when little-v’s case assigning potential is withdrawn in the passive, the recipient raises to subject.

Crucially, in the symmetrical passive languages, the two objects display the same case morphology in the active. There is no morphological contrast between the two objects on par with the dative-accusative contrast in German. Rather, both objects share the morphological encoding of direct objects. Suppose there were a language like Norwegian displaying the optionality in (54) but which looked morphologically like German, that is, in which the case that Appl assigns belonged to a different morphological paradigm than the case that little-v assigns. Call the former ‘dative’ and the latter ‘accusative’. In this case, assignment of case to the recipient by Appl would yield the dat-acc pattern with theme promotion to subject in the passive, just as seen in German. But assignment of case to the theme by Appl, as represented by the dotted lines in  (54), would yield a pattern in which the theme bears dative case and the recipient accusative. This pattern is not attested in any language to my knowledge. It follows that the putative optionality of upward vs. downward assignment of dative case by Appl is not attested in any language that actually differentially marks recipients and themes.

More generally, the notion that local case assignment to the recipient may be optionally withdrawn in languages like Norwegian is in conflict with Siewierska’s generalization. Siewierska observes that when themes are patient-encoded and recipients are morphologically distinguishable from themes, the differential encoding of recipients never alternates with patient encoding. But the characterization of Norwegian in (54) lets the local case of the recipient (assigned by Appl) alternate with patient encoding (accusative assignment by little-v). If this analysis is correct, it means that Siewierska’s generalization only holds in languages that do not morphologically differentiate the local case of the recipient and the case little-v assigns, i.e., that do not differentiate dative and accusative.

In terms of the analysis sketched in (54), the restriction required to enforce Siewierska’s generalization would take the form of the principle that Appl may assign case downward only if the case paradigm it assigns is identical to the case paradigm that little-v assigns. It is unclear what syntactic mechanism might be responsible for this principle, particularly in a framework where inflectional morphology is post-syntactic so that syntactic procedures operate independently of surface morphological facts (Halle and Marantz 1993).

In contrast to the case assignment approach described above, McGinnis (1998, 2001a, 2001b) and Anagnostopoulou (2003, 2005) argue that the facts of Norwegian and similar languages result from mechanisms available to these languages that obviate minimality. In principle, they claim, the mechanism that passes a theme up to subject position in the passive cannot reach past a recipient argument, when one is present. The recipient is a minimal candidate for raising to subject itself, and so intervenes in the Agree relation targeting the theme. What characterizes the languages that allow promotion of the theme to subject over the recipient in the passive is that in these languages, a predicate-internal escape hatch is available to the theme that is equidistant with the recipient to the probe. As a result, the order recipient>theme fails to ‘lock in’ in the domain with the escape hatch (ApplP here), so that the theme may be targeted by higher licensing operations.

Assuming that passivization affects the accusative-assigning potential of little-v, as these analyses do, the locality approach faces a case assignment dilemma that the case-based approach described above—problematic as it is for other reasons—does not have. Suppose in active double object constructions in Norwegian, little-v licenses accusative case on the recipient and Appl licenses accusative on the theme (as the dashed lines represent in (54)). Then withdrawal of the case licensing potential of little-v in the passive leads to promotion of the recipient to subject while the theme is still case licensed by Appl. But now consider the derivation of the theme passive. If the theme moves to an escape hatch above the recipient, for example an ‘outer’ specifier of ApplP, where it is closer to little-v, we would still expect its trace in [spec,VP] to receive accusative case from Appl, case-licensing the chain so formed. Then, withdrawal of little-v’s ability to license case should still only affect the recipient. If, on the other hand, the theme is for some reason no longer case-licensed by Appl when it moves to the escape hatch, but rather by little-v, to which it is now closer, we explain why it raises to subject in the passive but are left without a source of case for the recipient, which is not in the c-command domain of the other case licenser, Appl, nor accessible to little-v because of the now intervening theme.

However, in the analysis I have presented in Sect. 5, both instances of accusative case in active double object constructions in double accusative languages like Norwegian have the same source: default case-valuation. The ‘disappearance’ of accusative case on either a recipient or a theme in passive constructions need not be accounted for in terms of case withdrawal on this account. Rather, it is an epiphenomenon of the fact that in consequence of demotion of the agent into a PP, T probes beyond the agent and assigns nominative to one of the internal arguments prior to the point at which default case is assigned. The other argument receives accusative from the default mechanism as usual (if it has not already received dative by virtue of being in [spec,ApplP] in a dative language like German).

A locality approach with default accusative case explains why only double accusative languages like Norwegian can appear to violate Siewierska’s generalization, in the sense that either the recipient or the theme may raise to subject in the passive, giving the impression that the local case assigned to the recipient when the theme raises to subject alternates with accusative when the recipient itself raises. The reason is that there is in fact no local case assigner for the recipient in these languages. If there were, it might assign a case in a different paradigm than the source for accusative occurring on the theme. In the default case analysis, the two object cases have the same source—the default case mechanism—and therefore reflect the same morphological paradigm. As soon as a language has a distinct case assigner for recipients (Appl) than for themes (default case), then only the theme will promote to subject and the two cases may be morphologically distinct, as in German.^3^ This analysis therefore explains why a language like German cannot violate Siewierska’s generalization but a language like Norwegian can appear to do so. In the analysis presented here, the recipient never bears a local case in Norwegian. Rather, like the theme, it only ever bears default accusative.

Haddican and Holmberg (2019) argue that Anagnostopoulou’s (2003, 2005) locality analysis is based on a false empirical premise. Her analysis is motivated in the first instance by a correlation between the possibility of theme passivization over the recipient and the possibility of theme>recipient word order in actives, primarily across Swedish and Danish. The grammaticality of the theme passive in Swedish in (55a) (Anagnostopoulou’s 2003 example (182b), p. 124), she claims, is fed by the possibility of theme>recipient word order in the object shift example in (55b) (her example (187b), p. 127). In Danish, on the other hand, the ungrammaticality of the theme>recipient order in object shift contexts like (56b) (her example (186f), p. 127) blocks the derivation of the theme passive in (56b) (her example (186b), p. 126).^4^
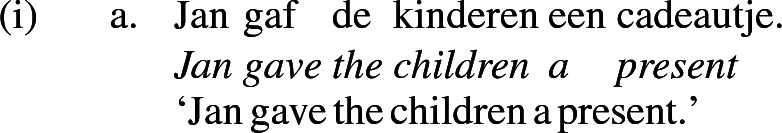

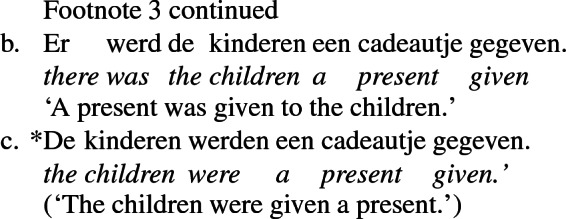


Haddican and Holmberg (2019) point out that although this correlation holds roughly across languages, it does not hold across individual speakers. In a large grammaticality magnitude estimation experiment on Norwegian, they found that the acceptability of inversion of the recipient and theme seen in (57) (Haddican and Holmberg’s (8b), p. 95) varied across speakers, and, crucially, individual speakers’ acceptance of (57) did not predict their acceptance of theme passivization illustrated in (53b). This should be impossible if the derivation of  (53b) prerequires the object inversion seen in (57). 
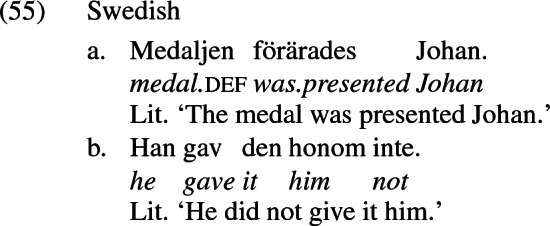


Further, object shift across negation is only available to unstressed pronouns in Mainland Scandinavian (Hellan and Platzack 1999, p. 127), while the theme passive seen in (53b) is available to full DPs. So even in Swedish, if the passive structure in (55a) were derived from the object shift structure illustrated in (55b), the impossibility of full DP themes in (55b) would be expected to prohibit full DP themes in (55a), contrary to fact. Further, in Swedish, where inversion of two objects seen in (55b) is more productive than in Norwegian, theme passivization is *less* productive: it is only available in the context of compound verbs containing an incorporated preposition such as *för* ‘for’ in (55a). Simplex verbs like *ge* ‘give’ only very marginally allow theme passivization, as (58) demonstrates (Holmberg and Platzack 1995, ex. 7.80a, p. 220). 
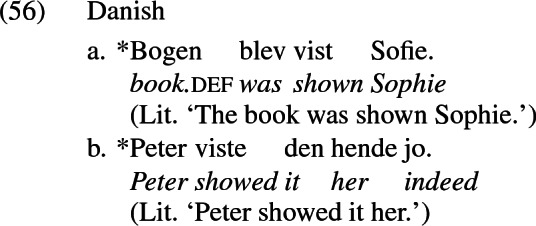


I take these observations to refute the idea that the predicate-internal inversion of the theme and recipient is a necessary precursor to theme passivization. They show instead that two different mechanisms are involved in the two constructions, and neither is a precursor to the other. But they do not militate against an analysis that relates the possibility of inversion in the passive to the (in)visibility of the recipient to the mechanism that raises the theme (either to a predicate-edge position in object shift contexts or the subject position in passives). Suppose raising of an object to subject in the passive in Norwegian is not restricted by minimality. That is, the nominative probe T may look down into its domain and establish an Agree relation with any DP with an unvalued case feature. In languages like Norwegian, where both objects are assigned case by default at spell-out, both objects are potential goals for T, as is of course the external argument in active constructions. As far as object case goes, standard English is like Norwegian. The difference between standard English and Norwegian, then, is that Agree is subject to minimality in English but not Norwegian, so that only the higher of the two objects can be promoted to subject in the passive, namely the recipient.^5^ Object shift in Norwegian is a separate operation that only targets pronouns. Whether it, too, is unrestricted by minimality varies from speaker to speaker, according to the experimental results of Haddican and Holmberg (2019).

It is crucial to this analysis of symmetric passivization that the external argument is not accessible to the default accusative case assignment mechanism. If the agent could receive default case in a language in which Agree is not sensitive to minimality, then T could probe past an agent in active constructions and assign nominative to an internal argument, triggering raising of the internal argument to subject, while the agent (and the other internal argument) receives default accusative case, a pattern that is to my knowledge not attested. Raising of an internal argument to subject is contingent on demotion of the agent, i.e., passivization. What blocks this pattern in the analysis I have proposed here is that default case is not available to an agent. The stipulation that default case is only available within the predicate makes it impossible for the agent to receive case if T assigns nominative to something other than the agent, unless the agent itself is demoted into a prepositional phrase, where it receives case from the preposition. Passivization is a way of giving case to an external argument when T targets an internal argument. This analysis therefore crucially requires that default accusative is restricted to a syntactic domain that excludes the external argument in [spec,vP], definable as v$'$, or the c-command domain of v, or as vP if specifiers are not dominated by the category they are specifiers of, as Kayne (1994) claims. Note that this way of blocking raising of internal arguments in active contexts is not easily reconcilable with the theory of dependent case marking as described by Marantz (1991), Baker (2015) and others. There, a DP receives accusative case if it is c-commanded by a clausemate DP, as objects always are but never subjects. But if an internal argument could raise past an external argument (in a language in which Agree is not restricted by minimality), that external argument would receive accusative case by the dependent case rule, being now c-commanded by the raised internal argument. The idea that accusative case is assigned by default in the domain to which the external argument is external correctly predicts that the external argument can never receive default accusative, and therefore must occur in a PP whenever an internal argument raises to subject, even in symmetric double object languages.

Although the analysis presented here does not make inversion under object shift a prerequisite for theme passivization, it is still more similar to the locality-based analysis of symmetric object languages than to the case-based approach. The idea is that languages vary parametrically in whether Agree relations are sensitive to minimality, that is, whether they can ‘see past’ a potential goal. I have endeavoured to show that a case-based solution essentially makes Norwegian an exception to Siewierska’s generalization, which in turn raises the question of why such putative exceptions only arise in double object constructions with symmetrical case marking, as in the mainland Scandinavian languages, and not in differentially marked constructions, as in German. According to the analysis proposed here, there are no exceptions to Siewierska’s generalization.

## Remarks on additional case frames

The analysis constructed above of symmetric and asymmetric double object languages is an analysis of change-of-possession ditransitives and how they differ from locative constructions. But other multiple object constructions are attested in German that display a wider variety of case frames than what I have discussed above. In addition to the dat-acc frame seen in change-of-possession constructions, which alternates with a periphrastic frame with *an* ‘at’, some verbs occur in a case frame in which the first object receives accusative and the second dative (59a), others with a frame in which the first object receives accusative and the second genitive (59b), and others with two accusative objects (59c) (examples cited from Alexiadou et al. 2014—their example (10), p. 8—who cite them from Beermann 2001; see also Lenerz 1977; Höhle 1982; Fanselow 1991, 2000; Haider 1993; Sternefeld 2006). 
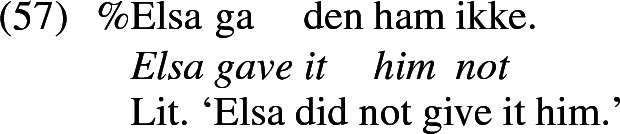


I assume these patterns represent syntactic articulations of the predicate distinct from the ApplP-VP complex that derives change-of-possession constructions. Assuming that each internal argument is generated as the specifier of a distinct head within the predicate—call the higher head $\hbox {V}_{{1}}$ and the lower $\hbox {V}_{{2}}$—and that the verb is introduced in $\hbox {V}_{{2}}$ and raises successively to $\hbox {V}_{{1}}$ and little-v, then [unterzieh-]$_{\hbox {v}_{2}}$ ‘subject’ assigns dative case to the specifier of $\hbox {VP}_{{2}}$ and [beschuldig-]$_{\hbox {v}_{2}}$ ‘accuse’ assigns genitive. Meinunger (2006) claims that ‘low’ datives as seen in (59a) are actually PPs whose preposition is incorporated into the verb, where it appears as a verb prefix (*unter-* ‘under’ in *unterzogen*, which is literally ‘pulled under’). Nothing I have claimed here is incompatible with this possibility.^6^ The accusative on the theme in (59c) could be assigned by the $\hbox {V}_{{2}}$ as well or by the default accusative assigning mechanism.

Although we are dealing here with multiple object verbs, which warrants an articulated predicate structure accommodating multiple objects in distinct syntactic projections, the dative DP in (59a) does not alternate with an *an*-phrase, as (60) demonstrates. This suggests that the alternation is sensitive not to dative case but to the context underlying change of possession, namely ApplP. I surmise that ApplP is not present in constructions like (59a) that do not support a periphrastic alternant. 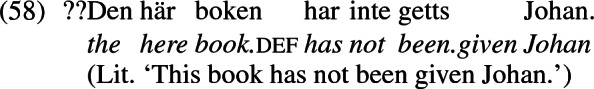


The absence of ApplP is presumably also at the root of the absence of an alternation with *an* in monotransitive constructions that take a dative object, like (61), which I assume has, like (59a) above, a dative-assigning $\hbox {VP}_{{2}}$ but no $\hbox {VP}_{{1}}$.^7^
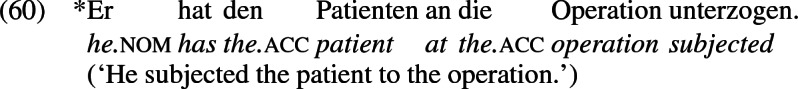


Another frame in which dative case occurs that is of interest for the present purposes is that of verbs that alternate with a form prefixed with *be-*. For example, *jemandem raten/someone.* dat *advise* ‘to advise someone’ alternates with *jemanden beraten/someone.* acc *advise*, with roughly the same meaning. Similarly, *jemandem drohen/someone.* dat *threaten* ‘to threaten someone’ alternates with *jemanden bedrohen/someone.* acc *threaten*, and *jemandem lauschen/someone.* dat *listen* ‘to listen intently to someone’ with *jemanden belauschen/someone.* acc *listen* ‘to eavesdrop on someone’, among other examples. This alternation affects a recipient in at least the case of *jemandem etwas schenken/someone.* dat *something.* acc *gift* ‘gift someone something’, which alternates with *jemanden beschenken/someone.* acc *gift* ‘gift someone (with something)’. Sometimes the DP that becomes accusative in the *be*-form occurs in a prepositional phrase in the base form, as in *etwas auf den Lastwagen laden/something.* acc *on the.* acc *truck load* and *den Lastwagen beladen/the.* acc *truck load* corresponding to ‘load something on the truck’ and ‘load the truck (with something)’ respectively, or *in dem Haus wohnen/in the.* dat *house live* and *das Haus bewohnen/the.* acc *house occupy* corresponding to ‘live in the house’ and ‘occupy the house’. For other verbs there is no case alternation, as in *jemanden grüßen/someone.* acc *greet* or *begrüßen* ‘greet someone’ and *jemanden schützen/someone.* acc *protect* or *beschützen* ‘protect someone’. While the variety of frames the bare verbs display and occasional deviations in meaning between the base and *be*-derivatives compromise a transformational analysis of the relation between the two verb forms, the *be*-forms at least all have in common that they do not license dative case. That accusative occurs instead is a natural consequence of the idea that accusative is a default object case on the present analysis. Are these examples, then, exceptions to Siewierska’s generalization?

Siewierska discusses languages in which the double object alternation is marked by a verbal affix, i.e., applicative constructions, citing the Indonesian example in (62) from Chung (1976, her examples (45a) and (46a), p. 54). The verbal suffix *-kan* occurs in the double object frame in (62b), complementary to the preposition *kepada* ‘to’ that marks the recipient in the periphrastic frame in (62a). 
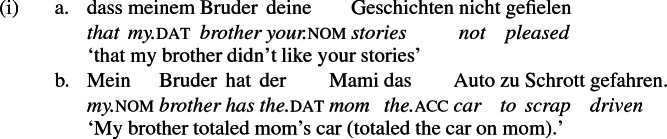


Siewierska endorses Baker’s (1988) claim that (62b) is derived from (62a) by incorporation of the preposition, so that the indirect object relation is marked in both examples, albeit differently. This situation bears an abstract resemblance to the *be*-verbs whose direct object occurs in a PP in the bare counterpart, such as *auf den Lastwagen laden* ‘load onto the truck’ and *den Lastwagen beladen* ‘load the truck’, which appears amenable to an analysis in which the preposition *auf* ‘onto’ disappears from its adnominal position and reappears in the form of the verb prefix *be-*, leaving its erstwhile DP complement to receive default accusative. In the cases where dative alternates with accusative in the *be*-counterpart, on some level dative itself manifests itself as the *be*- prefix instead.

Siewierska’s generalization is unaffected by these observations as long as it is understood to prohibit an alternation between dative and accusative *in the same context*. It is common, as in English and German, for a double object frame to alternate with a periphrastic frame in the same context, i.e., without any corresponding change in the morphology of the verb or other components of the sentence. But it is not observed that dative ever alternates with accusative in the same context. To the extent we find alternations between dative and accusative, such as the *be*-alternation in German, it is accompanied by a change in the context, in this case *be*-marking.

It is tempting to analogize the *be*-alternation to the applicative alternation in (62) by claiming that dative arguments of verbs like *raten* ‘advise’ (and for that matter accusative arguments of verbs like *grüßen* ‘greet’) are introduced by a covert preposition, and this preposition alternatively manifests itself as the verb prefix *be-*. However, allowing a null preposition to assign dative case would undermine Siewierska’s generalization unless restricted in crucial ways. We have observed that both accusative (in English) and dative (in German) DPs alternate with PPs (*to*- and *an*-phrases respectively). If a PP could be headed by a covert P that assigns dative, then in principle an alternation could arise between an accusative DP and a PP with a covert P assigning dative, which on the face of it would look like an alternation between accusative and dative. But Siewierska observes that this does not happen. This points to the conclusion that there are no covert prepositions.

The notion that dative is assigned by a covert preposition has been proposed to accommodate cases of what appears to be dative raising to nominative in passives, a situation which is puzzling from the perspective of Siewierska’s generalization. Larson (1988) cites the following paradigm in Japanese from Shimizu (1975) (Larson’s example (45), p. 365). The dative recipient in (63a) may appear as a nominative subject in the passive (63b) but not as an accusative object in the active, as (63c). 
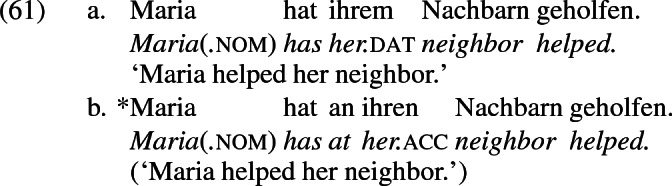


That dative in (63a) does not alternate with accusative, as (63c) shows, is in line with Siewierska’s generalization. But in order for the dative argument in (63a) to raise to nominative in (63b), it would have to shed its dative case in the course of the derivation. This is just what is impossible in German, as (6c) shows, for which reason dative is said to be an ‘inherent’ case in that language. Alexiadou et al. (2014) claim that dative case in the active (63a) is assigned by a covert preposition, which is incorporated into the verb in the passive, triggering raising of the DP to nominative. They further argue that German itself has raising of dative to nominative in passive constructions with the auxiliary *bekommen* ‘get’ as illustrated in (64b) (their example (22a), p. 12). They take the auxiliary *bekommen* ‘get’ to result from incorporation of a null preposition introducing the dative indirect object into the ordinary passive auxiliary *werden* ‘be’, resulting in promotion of the indirect object to subject. 
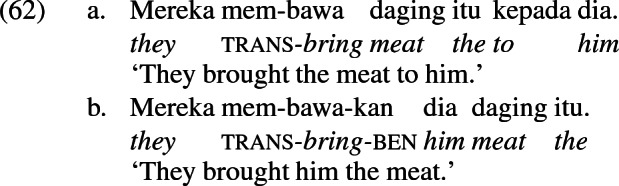


On this view, Japanese and German display raising of dative to nominative in passive contexts, but never of dative to accusative in active contexts. These considerations raise the question of why an alternation between dative case and a structural case is restricted to passive contexts. One answer that has a precedent in the literature on the alternation in (64) is that the cases of passivization in question do not actually involve raising of the dative argument to nominative, but rather base generation of the nominative recipient as an external argument. A sketch of an analysis along these lines is shown in (65). Abstraction over a covert dative pronoun in [spec,ApplP] shown there must be restricted to passive contexts and to only certain dative arguments, including recipients. The fact that the abstraction takes place at the level of vP, where passivization is morphologically cashed out, and that different dative arguments have different syntactic loci, as discussed above, makes this network of restrictions plausible. In German, only the auxiliary *bekommen* selects a predicate derived in this fashion; in Japanese this vP requires no special auxiliary. 
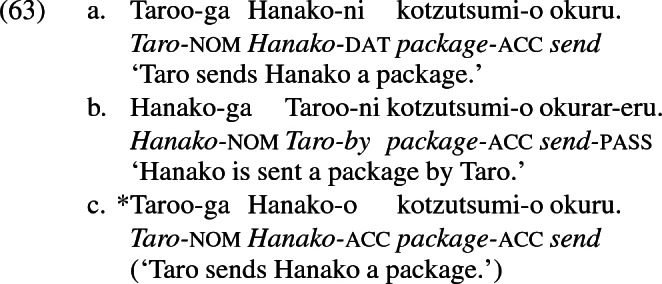


Evidence has been offered both for (Haider 1984, 1985; Vogel and Steinbach 1998) and against (Wegener 1985; Reis 1985; Fanselow 1987; Webelhuth and Ackerman 1994; Zifonun et al. 1997) non-derivational analyses of the relation between dative double object constructions and *bekommen* passives. The remarks above contribute to this debate by offering the observation that an analysis along the lines of (65) reconciles the apparent alternation between dative and nominative with the fact that no alternation between dative and accusative is observed: the dative and nominative variants are not actually transformationally related. This approach eschews null prepositions, which is advantageous since these potentially undermine Siewierska’s generalization. Whether this approach will stand up to further scrutiny, and if not, how a true dative-nominative alternation can be reconciled with Siewierska’s generalization, remains to be seen.

## Conclusion

This article has investigated the source of a cross-linguistic gap noticed by Anna Siewierska, that no language displays an alternation between dative and accusative encoding of recipients in double object constructions. I have claimed that this gap implicates a cross-linguistic universal that dative case cannot be withdrawn in a given syntactic context. Yet, both dative and accusative recipients alternate with periphrastic encoding marked by a preposition, in which case the recipient DP receives the case assigned by the preposition. This, I have argued, results from the possibility of generating the recipient as a PP in the recipient theta position, on par with certain analyses of passive constructions. German has a reasonably productive double object alternation, where dative recipients alternate with periphrastic encoding just like English accusative recipients do, demonstrating that the alternation between the double object frame and the periphrastic frame is not sensitive to the case of the recipient. Lastly, I have claimed that symmetrical passivization, in which a theme may be raised to subject over a recipient, does not indicate that the recipient bears (unmarked) dative case in that configuration, since then the possibility of raising the recipient to subject (which is available in symmetrical languages) would represent an alternation between dative encoding of the recipient and patient encoding—the encoding that is withdrawn or not assigned in the passive. That is just the situation that Siewierska shows is not attested. I claim instead that what characterizes the symmetrical languages is a lack of strict minimality in the licensing of nominative case. The system proposed here ensures that no language displays an alternation between dative and accusative recipients.

## Footnotes

1 Sometimes prefixed and unprefixed forms of a verb admit different complement frames, as in the case of *weiter-* or *zurückgeben* ‘pass on’ or ‘give back’ (9), which admit the periphrastic frame that underlying *geben* ‘give’ excludes (22b). The data in (7)–(21) contain both prefixed and unprefixed verbs that display both frames, indicating that verb morphology does not strictly determine a verb’s complement frame. Accordingly, the analysis I present in Sect. 5 does not connect frame selection with verb morphology. However, Susi Wurmbrand (p.c.) points out a subgeneralization about the verb prefix *ver-*: if a bare verb alternates with a *ver*-prefixed counterpart under roughly the same meaning, they divide the labor of licensing the two frames between them: *V* occurs with the double object frame and *ver-V* with the periphrastic frame, hence the complementarity between (22) and (i) below. Verbs prefixed with *ver-* that allow both frames either have a bare counterpart with a different meaning (e.g., *verkaufen* ‘sell’ in (7) vs. *kaufen* ‘buy’) or do not have a bare counterpart at all (e.g., *verabreichen* ‘administer’ in (17)). The complementarity between (22) and (i) therefore appears to be a blocking interaction between derivationally related lexical items. Footnote 1 continued

2 For German, this requirement should not be construed as requiring there to be a highest DP. German allows ‘impersonal’ passives of intransitive verbs, as in (i). In these cases, T fails to find a highest DP to agree with. No nominative element appears and default agreement obtains.

3 Nothing in this analysis *requires* the two cases to be morphologically distinct. In fact, standard Dutch does not morphologically distinguish the case of the recipient from that of the theme, yet exhibits the same passivization pattern as German, where only the theme may be promoted to subject in the passive, as the pattern in (i) illustrates (Broekhuis et al.’s 2015 example (115), p. 444). These data lend themselves to an analysis that gives local dative case to the recipient and default accusative to the theme, though the two are not morphologically distinguished.

4 Anagnostopoulou cites (55a) from Holmberg and Platzack (1995, ex. 7.75, p. 218), (55b) as a personal communication from Anders Holmberg parallel to similar data in Hellan and Platzack (1999, p. 131), (56b) from Vikner (1989, ex. 40b, p. 150), and (56b) from Vikner (1989, ex. 45f, p. 151).

5 A reviewer asks what might determine the choice of recipient or theme raising to subject in passives in a language in which Agree is not restricted by minimality, and whether the factors at work are similar to the factors at work in the double object alternation. Corpus research by Bresnan et al. (2007), Rappaport Hovav and Levin (2008) and Bresnan and Nikitina (2010) demonstrates that the double object alternation in English is generally put to use to align the linear order of arguments with a hierarchy of features in which pronouns supercede definites/animates, which supercede indefinite/inanimates, which supercede non-referential material like idiom chunks. Object shift in Mainland Scandinavian is clearly subject to a grammaticalized high definiteness requirement, which, as I mentioned above, raising to subject is not. I am not familiar with similar work on recipient vs. theme raising in symmetric passive languages. Standard examples like (53b) suggest that animacy is not a crucial factor either. The analysis I have proposed Footnote 5 continued here makes the choice of recipient or theme raising in passive truly optional, but we might still expect a preference for minimality-respecting transformations as well as discourse pragmatic factors to play a role in facilitating one or the other in a given speech context, as they do in the double object alternation.

6 It is worth pointing out in this connection that the dative DP in (59a) translates into English as a *to*-phrase, and therefore the idea that dative DPs in German correspond to *to*-phrases in English is correct in some contexts. But the correspondence only holds for dative objects of verbs like *unterziehen* ‘subject s.o. to s.t.’, *zuschreiben* ‘attribute s.t. to s.o.’ and others, all of which show basic acc-dat word order, unlike the basic dat-acc word order of change-of-possession constructions. These constructions therefore do not undermine Siewierska’s point that dative recipient DPs in German are not on par with *to*-phrase recipients in English. The data reviewed here indicate instead that German *an*-phrase recipients are on par with English *to*-phrase recipients, though *to* may correspond to dative in other contexts.

7 Two additional case frames that warrant further investigation are the frame associated with dative experiencers and that associated with dative ‘external possessors’. The phrase *meinem Bruder* ‘my.dat brother’ in (ia) (den Besten’s 1985 example (25b), p. 32) is interpreted as an experiencer. Such datives are argued to be base generated above the (nominative) theme in den Besten (1985), Haider (1993, 2006), Wurmbrand (2006), and elsewhere. As such, they bear a resemblance to the present analysis of recipients in double object constructions. Yet, these datives do not alternate with *an*-phrases, a fact that is presumably related to the fact that they bear a different theta role, even if the syntactic structure they are generated in is similar to that of recipients. Oblique experiencers are perhaps specifiers of a ‘flavor’ of Appl distinct from that which licenses possessors, one that does not have a periphrastic alternant. Similarly, the phrase *der Mami* ‘the.dat mom’ in (ib) (Lee-Schoenfeld’s 2006 example (2a), p. 104) is interpreted as an affected ‘external possessor’ (of the car in question) yet, like an experiencer, cannot be expressed in an *an*-phrase. Some analyses of this phenomenon generate the external possessor as an ‘internal possessor’, i.e., as a specifier of the corresponding possessee (*das Auto* ‘the.acc car’ in (ib)) (Szabolcsi 1984; Landau 1999; Lee-Schoenfeld 2006). This view explains why they do not alternate with *an*-phrases: only specifiers of ApplP do so. Other analyses make them specifiers of their own light verb projection (Pylkkänen 2002, 2008; Cuervo 2003a; Brandt 2006, to mention a few). McFadden (2006) assimilates all dative arguments to an underlying ApplP. If this is the right approach, projections hosting affected possessors must also represent a different flavor of Appl than what is found in change-of-possession constructions.
